# Repairing tendon-exposed wounds by combing the Masquelet technique with dermoplasty

**DOI:** 10.3389/fsurg.2022.995316

**Published:** 2022-11-02

**Authors:** Jiangling Yao, Yunfu Zeng, Jian Yang, Qian Wu, Liying Chen, Linyang Zheng, Rong Wang, Hengjie Zhu, Hongwang Cui, Yuesheng Huang, Shaowen Cheng

**Affiliations:** ^1^ Emergency and Traumatology Department, The First Affiliated Hospital of Hainan Medical University, Hainan Medical University, Haikou, China; ^2^ Key Laboratory of Emergency and Trauma Ministry of Education, Hainan Medical University, Haikou, China; ^3^ Academy of Pediatrics, Hainan Medical University, Haikou, China; ^4^ Institute of Wound Repair and Regeneration Medicine, Southern University of Science and Technology School of Medicine; Department of Wound Repair, Southern University of Science and Technology Hospital, Shenzhen, China

**Keywords:** exposed tendon, wounds, skin grafting, Masquelet, gentamicin bone cement

## Abstract

**Background:**

Wound repair is a new field that has emerged in China in the last 5 years. Exposed tendon wounds are one of the most common problems faced in wound treatment today, as the poor blood supply leads to low survival rates of skin grafts. This paper explores the feasibility of applying the Masquelet technique to repair tendon-exposed wounds.

**Method:**

We examined 12 patients with tendon-exposed wounds, 5 males and 7 females, from January 2021 to November 2021, including 2 patients with post-traumatic wounds, 8 diabetic patients with dorsal wounds, and 2 patients with various chronic infections. The Masquelet technique was employed to treat these wounds. The wound surface was sealed with antibiotic bone cement to form an induction membrane, the cement was removed after 3–4 weeks, and the wound was repaired with skin grafts to observe survival, appearance, texture, healing, and related functions.

**Results:**

All wounds were covered with antibiotic bone cement, and after 3–4 weeks, an induction membrane was applied, and in 10 out of 12 patients, full-thickness skin grafts were applied, and the patients survived. However, in 2 patients, the skin became partially necrotic, but these patients recovered by changing medications.

**Conclusion:**

The current study found that direct skin grafting may effectively treat exposed tendon wounds once the Masquelet approach generates the induction membrane. Further, this method is less difficult, less expensive, and easier to care for the procedure that deserves to be used more frequently.

## Introduction

The number of acute wounds and chronic conditions based on chronic wounds (such as diabetic foot, pressure ulcers, and venous ulcers) is rising quickly due to rapid economic development and the population's accelerated ageing. Skin and soft tissue loss in the limbs are the most prevalent, typically resulting in tendon-exposed wounds (1). For tendon-exposed wounds, we often use flap prostheses or skin grafts after long-term drug replacement, but flap prostheses are difficult, and manipulation is challenging (2). Some wounds require secondary flap prostheses because the thick flap partially affects appearance and function. Poor vascular and skin conditions can lead to flap necrosis, making this treatment option rarely available. In addition, long-term drug treatment can lead to long-term exposure to the tendon and corresponding functional impairment due to tendon necrosis, finally seriously affecting the patient's quality of life (3).

In recent years, some scholars have applied antibiotic-containing gentamicin bone cement to treat various wounds with some success. However, there are no reports of repair of tendon-exposed wounds after covering them with antibiotic-containing gentamicin bone cement, followed by skin grafting directly to the guiding membrane (4). From January 2021 to November 2021, this study successfully repaired tendon-exposed wounds using this technique in 12 patients. These wounds underwent prompt and complete cleaning before being sealed with gentamicin bone cement, which was removed 3–4 weeks later. An induced membrane started to develop. Thick skin grafts were used to treat the wounds directly, and they were successfully repaired.

## Methods

### Patients

Twelve patients (5 males and 7 females) ages 47–89 years (mean age 68.75) were recruited for the current study. Among all selected patients, 2 showed post-traumatic wounds, 8 diabetic foot wounds, 2 with other chronic infected wounds, 10 with ground and foot wounds, and 2 with dorsal hand wounds. The wound defects range from 3 cm × 2 cm to 18 cm × 12 cm, and disease duration ranges from 1 to 4 months.

After anaesthesia, disinfection, and draping, infected necrotic tissue was completely removed, and the wounds were cleaned until fresh blood drained from their wounds. The wound was cleaned with 3% hydrogen peroxide, diluted iodine, and normal saline. The wound blood was stopped by electrocoagulation or fine wire ligation and was checked and re-cleaned if necessary.

### Treatment method

Gentamicin Bone Cement (Depuy Synthes, USA) powder cement (40 g) was taken and thoroughly mixed. The polymethyl methacrylate reagent (Depuy Synthes, USA) was added and mixed to create a paste. Following the drawing step, it was thinly tiled to fit the size and shape of the wound before being covered and moulded. Before the gentamicin bone cement hardened, a 1.5 mm Kirschner wire was used to drill holes in its surface. In order to cover the cement surface, sutures were used to attach the gentamicin bone cement to the wound surface. Postoperative anti-infective care involving intravenous static antibiotics was necessary.

One debridement was performed 5–7 days after admission and covered with gentamicin bone cement after 1–2 thorough debridements. Every 2 days after Stage I surgery, the wound dressing was changed. The wound exudate and redness and swelling around the wound were observed. Two weeks later, the gentamicin bone cement was removed. The physician monitored infection control and new granulation growth, collected wound secretions, and performed bacterial culture and drug sensitivity testing. Depending on the wound's size and the granulation's growth, the decision was made to use relaxing sutures, subsequent drug replacement, or skin grafting. If was no improvement in wound infection or only a small amount of fresh granulation tissue has begun to grow, another debridement and antibiotic gentamicin bone cement can be used. Suppose there was no bacterial infection and a large amount of granulation tissue begins to grow in the wound culture. In that case, the physician should observe a smooth guiding membrane formed on the tendon and wound, perform a full-layer skin graft, and secure the plaster externally using “packing” combined with a compression bandage to prevent skin loosening and necrosis due to activity. The compression bandages were removed 7–10 days after surgery. If some skin grafts were not viable, the dressing was changed every 2 days, and recombinant bovine basic fibroblast growth factor (Essex Bio-Technology, Hong Kong) gel was applied externally until the wound healed.

The Masquelet technique must be used to treat these wounds. This method involves sealing the wound with gentamicin-containing bone cement to produce an induction membrane, removing the cement after 3–4 weeks, covering the wound with a direct skin graft, and monitoring the grafted skin's survival, shape, feel, and wound healing.

### Hematoxylin-eosin staining

Human wound tissues covered with gentamicin bone cement were collected during surgery, fixed with 4% formaldehyde, embedded in paraffin, and then cut into 8 μm-thick serial sections. The sixth layer of each section was selected for hematoxylin-eosin (HE) staining, and the images were observed and recorded under a light microscope with a 40× objective.

## Results

After complete debridement, antibiotic gentamicin bone cement was applied to all wounds, and in 3–4 weeks, an induction membrane developed. The two patients with partial necrosis of the grafted skin recovered with the replacement of dressings, and all 10 of the 12 patients who underwent all-layer skin grafting survived.

### Typical case 1

**Figure 1:**
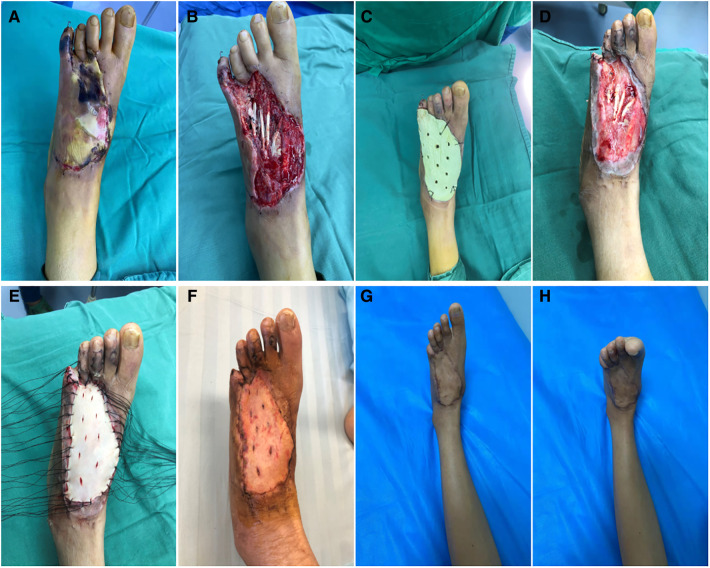
(**A**) Pre-operative. (**B**) Exposed tendons. (**C**) Gentamicin bone cement covers the wound. (**D**) Induction of membrane formation. (**E**) After implant surgery. (**F**) Fourteen days after implant surgery, the dressing was removed. (**G,H**) Three months after implant surgery.

A 47-year-old female patient was admitted to our hospital with “left leg pain from a car accident for the past 5 days”. She had a normal medical history, and the left sole was identified upon admission. The patient was treated with wound sealing with debridement + gentamicin bone cement coverslip + VSD for skin liquefaction necrosis after admission while receiving a nerve block (Figure 1A). The injury was on the left dorsal foot. In the surgery, liquefied necrotic tissue was excised, a culture specimen was obtained, and an irregular wound measuring approximately 8.0 cm × 10.0 cm was formed, exposing two to five extensor toe tendons (Figure 1B). The wound surface was covered and shaped. The gentamicin bone cement surface was perforated with a 1.5 mm Kirschner wire before the cement solidified, and sutures were used to secure the gentamicin bone cement to the wound surface (Figure 1C). On postoperative day 21, the gentamicin bone cement with antibiotics was harvested. It was evident that an inductive membrane had formed on the surface of the tendon (Figure 1D). Therefore, a full-layer skin graft was performed, and the entire wound was wrapped with packing and compression bandages (Figure 1E). The skin graft survived 14 days after skin grafting (Figure 1F). At the 3 month postoperative follow-up, the affected area had recovered well in appearance and function (Figures 1G,H).

### Typical case 2

**Figure 2:**
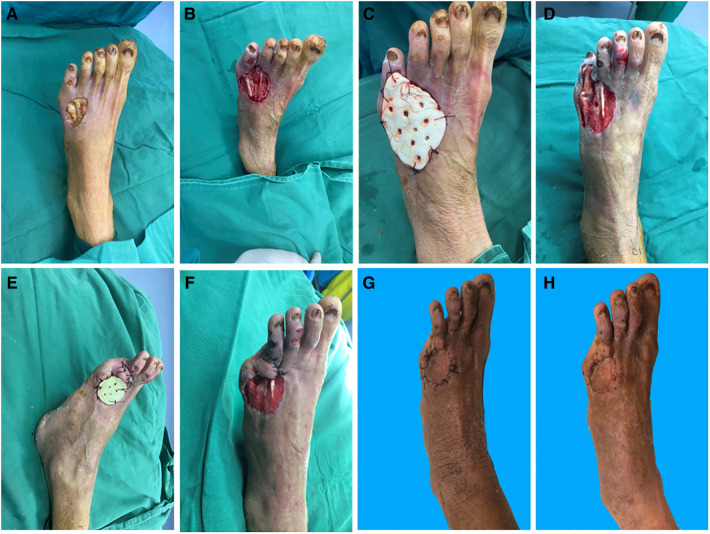
(**A**) Pre-operative. (**B**) Exposed tendons. (**C**) Gentamicin bone cement covers the wound. (**D**) Induction of membrane formation. (**E,F**) Before implantation. (**G**) Fourteen days after implant surgery. (**H**) One month after implant surgery.

A 68-year-old woman was admitted to our hospital with a skin ulcer and pus on the left foot that had persisted for over a month and worsened over a week in the affected area. The patient had a history of diabetes for over 3 years. She used insulin for blood glucose control and did not regularly measure blood glucose levels. On admission, there was a 3 cm × 4 cm skin defect on the anterior edge of the right instep with erythema and swelling of the surrounding tissue. Numerous purulent secretions with exposed tendons were noted (Figure 2A). After appropriate testing, the patient underwent surgery for wound closure with debridement of the left dorsum + gentamicin bone cement covering + vacuum aspiration under a nail block of the left dorsum 7 days after admission. After initial thorough debridement, a 5 cm × 6 cm wound was formed, the tendon was exposed, a specimen was taken and cultured (Figure 2B), and then gentamicin bone cement was sutured and fixed to the wound (Figure 2C). On postoperative day 30, the gentamicin bone cement with antibiotics was removed. It was evident that a layer of induction membrane had formed on the surface of the tendon (Figure 2D). During the pre-implant surgery, a substantial amount of fresh granulation tissue was found encircling the exposed tendon and indicating the attachment of a new induction membrane (Figures 2E,F). A full-layer skin graft was placed. The skin graft began to survive after 7 days (Figure 2G). Finally, the wound healed well after 1 month of follow-up (Figure 2H).

## Discussion

Tendons are a special type of tissue in which the tendon receives little blood supply, except at the bony ends, but the tendon depends on the surrounding interstitial fluid for its survival (5). When the tendon is exposed, the tendon membrane can become deficient due to trauma or infection. The wound is prone to bacterial contamination, so the tendon must be repaired and covered. Otherwise, the wound can easily lead to serious complications such as tendon wound infection and ischemic tendon necrosis (6). Some patients have complex conditions in clinical practice, such as unstable general conditions due to multiple underlying medical complications, combined wound infections that prevent thorough debridement, too large wound defects, and limited flap zone conditions. Stage-I repair of the exposed tendon cannot be performed, so only the wound can be temporarily placed and await secondary repair (7). The conventional approach to treating putting-aside requires debridement followed by a dressing change with a sterile dressing, which takes time and saves just a small amount of the tendon. Vacuum drainage has been increasingly popular among medical practitioners in recent years. Although tendon yellowing, degeneration, and necrosis are seen after generating VSD, it is generally utilised for various acute and chronic wounds (8).

**Figure 3:**
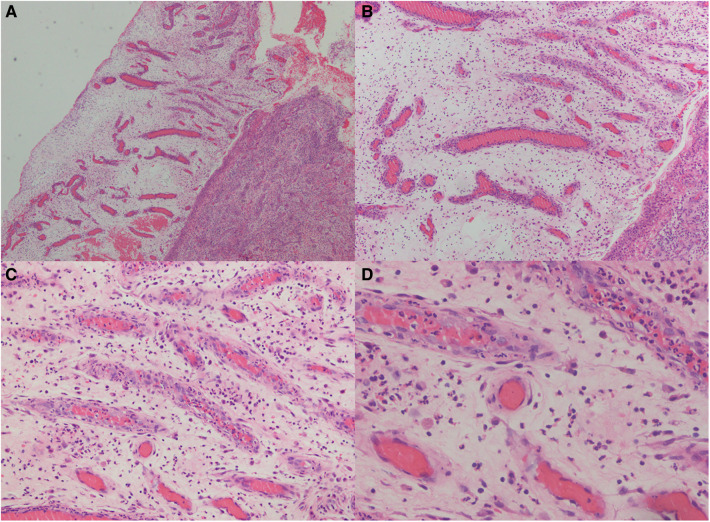
Hematoxylin and eosin (HE) staining of the induced membrane tissue at different magnifications. The developed membranes were widely dispersed and had considerable neovascularization, which increased the likelihood that the flap transplant would survive (magnification from **A–D** are: 40x, 100x, 200x, 400x).

The impact of the wound microenvironment on wound repair is progressively becoming understood because of developments in histology and cell function (9). Masquelet technique, also called induced membrane technique, is an emerging field based on human foreign body reaction and bone regeneration capabilities. It was first reported by French scholar Masquelet in 1986 as a treatment for large bone defects (10). The thickness of the induction membrane and synovium created by Masquelet is almost the same, ranging from 0.5 to 2.0 mm. There are small blood vessels on the induction membrane (Figure 3), and immunohistochemical analysis showed that the induction membrane capsule is composed of type I collagen, its inner layer is composed of synovial epithelial cells, and its outer layer is rich in fibroblasts and myofibroblasts. At the same time, the inductive membrane capsule is not only densely populated with small blood vessels in the long axis direction. However, it can also secrete VEGF, TGF-β1, and BMP-2 to contribute to local vessel formation (11–13). To date, the Masquelet technique has been used in, but not limited to, the reconstruction of large bone defects (14), the treatment of infected femoral defects after trauma (15), the reconstruction of finger bones (16), chronic osteomyelitis (17), the reconstruction of the chest wall (18), and in the treatment of wounds. Gentamicin bone cement is applied to the wound to form an induction membrane. An inflammatory reaction sets the stage for wound healing, followed by granulation and tissue growth. Angiogenesis is crucial for tissue repair at this point. As a result, the author hypothesises that gentamicin bone cement also treats wounds infected with soft tissue (19, 20).

By collecting clinical data, the author found that Masquelet technology can temporarily cover a wound. Covering with gentamicin bone cement creates a separate space, isolates it from the external environment, avoids exogenous infection, and seals the wound to promote the metabolism of wound tissue fluids (21). Keeping the wound moist is beneficial for epidermal cell regeneration, activation of growth factors, and promotion of vasculature and granulation. It is especially important for the nutritional supply to the tendon, i.e., creating a physiological survival environment for the tendon. In other words, it is similar to creating a condition after flap repair (22). A typical colour characterises the covered tendon, and an inductive membrane gradually develops. This offers favourable circumstances for subsequent tendon-exposed wound healing. In addition, antibiotic gentamicin bone cement works well as a temporary filler for the space left behind after the removal of necrotic tissue, lowering the bacterial load of the wound, preventing further tendon injury, delaying tendon and wound healing, lowering the need for intravenous antibiotics, and lowering toxic effects on the body's liver and kidney function (11, 23). Stage-1 Thorough debridement is the cornerstone of successful follow-up surgery. Local infection should be prevented and treated with gentamicin bone cement. However, thorough debridement is a prerequisite for successful follow-up surgery, and ultimately care must be taken to protect the induced membrane during surgery (24). Stage-2 When removing gentamicin bone cement during surgery, the gentamicin bone cement must be gently removed, and the induced cement integrity must be maintained; 1–2 weeks is a period of thorough debridement, infection control, and systemic remediation. The vascular activity of the induced membrane peaks at 2–4 weeks. Based on the clinical data collected, the author has determined that approximately 3 weeks postoperatively is the optimal time for induced epithelial grafting, with better results.

Debridement of the wound and antibiotics to prevent infection and lessen pressure on the foot are the current gold standard of treatment for diabetic foot ulcers. Neuropathic, ischemic, and neuro-ischemic diabetic foot ulcers can be distinguished at this stage, with neuro-ischemic being the most prevalent. The ulcerative lesions are made worse by the absence of neurotrophic support and the accompanying vascular disease, resulting in amputation and a bad prognosis for the patient. Current treatment options include interventional therapy, antibiotic therapy, hyperbaric oxygen therapy, electrical stimulation therapy, multiple dressings for wounds, vacuum sealing drainage (VSD), biological therapy, surgical debridement, skin grafting, flap grafting, joint fusion, etc. The main reason for the differences in the treatment of tendon outgrowth due to diabetic foot ulcers is the differences in the bacterial species of the wounds, especially when it comes to fungal infections similar to MASA and other drug-resistant bacteria. On the other hand, age and diabetic neuropathy cause the elderly to be more prone to gangrene and even amputation. The length of hospitalization for tendon exenteration compared to ordinary diabetic foot ulcer wounds and the economic pressure leading to treatment interruptions in the patient's treatment process are also important factors in the difference in treatment outcome.

The first patient in this study has experienced trauma, while the second patient has a diabetic foot ulcer. Poor blood perfusion decreased blood flow to the limb tissues, the patient's obesity or poor health status, persistent, localised pressure on the torso, and a considerable decline in skin elasticity and thickness distinguish diabetic foot wound patients from those with traumatic ulcers. As a result, tendon outgrowth brought on by trauma heals more quickly than tendon outgrowth brought on by a diabetic foot.

## Limitations

This study discovered that the Masquelet technique and skin grafting procedures do not have complete efficacy metrics for tendon debridement wounds. However, we noticed an increase in wound granulation and a percentage reduction in the size of the wound. This study did not employ comparison studies or other research techniques due to the brief observation duration. More samples are required to establish the efficacy of the Masquelet technique and skin grafting for the treatment of exposed tendon wounds due to the small sample size of this study.

## Conclusion

In summary, Masquelet technology can, in essence, create a transparent guiding membrane over the wound, create a favourable angiogenic environment, cover exposed tendon wounds, protect tendons, prevent infection, lessen the overuse of antibiotics, decrease dead space, and encourage granulation and tissue formation. On the other hand, when used with skin grafting, it may successfully cover acute, chronic, and exposed tendons, producing better and faster results, making it worthwhile to promote and use.

## Data Availability

The original contributions presented in the study are included in the article/Supplementary Material, further inquiries can be directed to the corresponding author/s.
